# Dynamics of SIN Asymmetry Establishment

**DOI:** 10.1371/journal.pcbi.1003147

**Published:** 2013-07-11

**Authors:** Archana Bajpai, Anna Feoktistova, Jun-Song Chen, Dannel McCollum, Masamitsu Sato, Rafael E. Carazo-Salas, Kathleen L. Gould, Attila Csikász-Nagy

**Affiliations:** 1 The Microsoft Research-University of Trento Centre for Computational Systems Biology, Piazza Manifattura 1, Rovereto, Italy; 2 Howard Hughes Medical Institute and Department of Cell and Developmental Biology, Vanderbilt University, Nashville, Tennessee, United States of America; 3 Department of Microbiology and Physiological Systems and Program in Cell Dynamics, University of Massachusetts Medical School, Worcester, Massachusetts, United States of America; 4 Department of Biophysics and Biochemistry, University of Tokyo, Tokyo, Japan; 5 Department of Life Science and Medical Bioscience, Waseda University, Tokyo, Japan; 6 The Gurdon Institute, University of Cambridge, Cambridge, United Kingdom; 7 Department of Computational Biology, Research and Innovation Center, Fondazione Edmund Mach, San Michele all'Adige, Italy; 8 Randall Division of Cell and Molecular Biophysics and Institute for Mathematical and Molecular Biomedicine, King's College London, London, United Kingdom; University of Illinois at Urbana-Champaign, United States of America

## Abstract

Timing of cell division is coordinated by the Septation Initiation Network (SIN) in fission yeast. SIN activation is initiated at the two spindle pole bodies (SPB) of the cell in metaphase, but only one of these SPBs contains an active SIN in anaphase, while SIN is inactivated in the other by the Cdc16-Byr4 GAP complex. Most of the factors that are needed for such asymmetry establishment have been already characterized, but we lack the molecular details that drive such quick asymmetric distribution of molecules at the two SPBs. Here we investigate the problem by computational modeling and, after establishing a minimal system with two antagonists that can drive reliable asymmetry establishment, we incorporate the current knowledge on the basic SIN regulators into an extended model with molecular details of the key regulators. The model can capture several peculiar earlier experimental findings and also predicts the behavior of double and triple SIN mutants. We experimentally tested one prediction, that phosphorylation of the scaffold protein Cdc11 by a SIN kinase and the core cell cycle regulatory Cyclin dependent kinase (Cdk) can compensate for mutations in the SIN inhibitor Cdc16 with different efficiencies. One aspect of the prediction failed, highlighting a potential hole in our current knowledge. Further experimental tests revealed that SIN induced Cdc11 phosphorylation might have two separate effects. We conclude that SIN asymmetry is established by the antagonistic interactions between SIN and its inhibitor Cdc16-Byr4, partially through the regulation of Cdc11 phosphorylation states.

## Author Summary

Rod shaped fission yeast cells, as the name suggests, divide by medial fission. The proper timing of this cytokinesis and septation event is controlled by a signaling pathway called the Septum Initiation Network, or SIN. The SIN is activated only after chromosomes start to separate in anaphase. At this stage, the two daughter spindle pole bodies (SPBs - the yeast analog of centrosomes) have separated and are on their way to the distant tips of the cell. SIN components are localized to SPBs, but the SIN is active only at one SPB, while the Cdc16-Byr4 complex keeps the SIN inactive at the other SPB. This asymmetric activation of the SIN is important for proper cell division as perturbation of this can lead to appearance of multiple septa or total lack of septation. The molecular mechanisms that are important for asymmetry establishment are emerging, but we lack a complete picture. Here we develop computational models to capture the dynamical features of asymmetry establishment and to determine the key components and interactions that are needed for proper asymmetric SIN activation. Our predictions and their experimental tests reveal some basic features of the system and highlight missing points in our knowledge.

## Introduction

Cell division is a fundamental and conserved process in all eukaryotes. The fission yeast *Schizosaccharomyces pombe* has already proved to be a very simple yet interesting model system to study and analyze eukaryotic cell division [1]–[3]. The onset of cytokinesis must be tightly coupled to the completion of mitosis for proper segregation of chromosomes into two daughter cells. In fission yeast, the initiation of cell division is controlled by a conserved signaling pathway known as the Septation Initiation Network or SIN [4]–[9]. Regulation of the SIN happens at the spindle pole bodies (SPBs) of fission yeast cells, where the scaffold proteins Cdc11 and Sid4 localize the rest of the molecules in the network [10], [11]. At the top of the pathway sits the GTPase Spg1, which controls a protein kinase pathway that triggers actomyosin ring contraction and positively regulates septum formation [12]. The Cdc16-Byr4 GAP complex negatively regulates SIN by inactivating Spg1 [13]. During interphase Cdc16-Byr4 keeps Spg1 inactive, but in metaphase the GAP complex is removed from SPBs, allowing the accumulation of the Cdc7 kinase to both SPBs [14]. As cells enter into anaphase Spg1-GTP gets hydrolyzed by the appearing Cdc16-Byr4 complex and Cdc7 disappears from the old SPB (that was existing already in the mother cell [15]). At the same time Cdc7 level rises at the new SPB with Spg1 remaining in GTP bound form and without the presence of Cdc16-Byr4 [16]–[18]. Such asymmetric segregation of the active SIN (Spg1-GTP and Cdc7), and its inhibitory complex (Cdc16-Byr4) is essential for proper activation and eventual inactivation of the SIN [19].

The role of this asymmetry was investigated recently and it was found that phosphorylation-dephosphorylation events on the scaffold protein Cdc11 by the downstream SIN kinase Sid2 and the SIN Inhibitory Phosphatase complex (SIP) play important roles in the establishment of SIN asymmetry between SPBs [20], [21]. Still the detailed molecular mechanisms that ensure efficient and fast asymmetry establishment and turning off of SIN activity after cell division is not well understood [19]. Here we develop mathematical models of increasing complexity to understand what basic features such an asymmetry generating system might contain and what known interactions of SIN and its regulators might be important for such features.

Mathematical modeling was already successfully used to capture dynamical features of the timing of SIN activation [4] and the orthologous pathway in budding yeast was also investigated this way [22]. Future experimental and modeling work will be needed to merge all knowledge on the spatio-temporal regulation of the SIN into a detailed model that could capture all molecular regulatory interactions in a quantitative way. Here we make the first steps on this line by focusing on the dynamics and regulation of SIN asymmetry establishment in a qualitative fashion.

## Results

### A minimal model of asymmetry establishment between two SPBs

**Figure 1:**
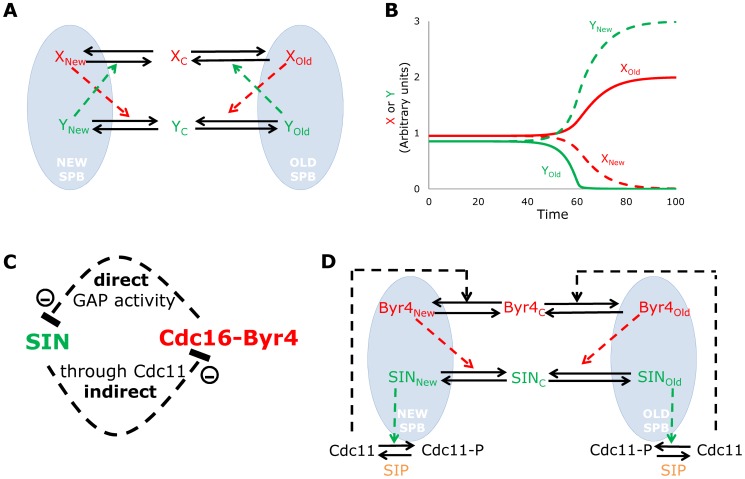
A minimal model for SIN asymmetry establishment. (**A**) Direct antagonistic interactions between molecule X and Y at the two SPBs. Both molecules induce the removal of the other from the SPB they are both bound. Solid lines are transitions, dashed arrows show catalytic effects. (**B**) A less than 0.1% difference in the SPB binding rates or in initial conditions (not shown) can induce quick asymmetry establishment. Solid lines for molecules at old SPB, dashed lines for molecules at new SPB, time is in arbitrary units. (**C**) The proposed antagonistic double-negative ( = positive) feedback between SIN components and Cdc16-Byr4. (**D**) Merging ideas from panels A and C to create a minimal molecular model of asymmetry establishment.

The minimal mechanism whereby asymmetry could be established between the two SPBs needs to contain some type of positive feedback loop, which involves a non-linear step [23], [24]. These are the minimal requirements to reach bistability, where one SPB ends up in a steady state with active SIN, while the other settles in an inactive SIN steady state. The two SPBs communicate through releasing and anchoring molecules from the cytoplasmic pool, thus these binding-unbinding steps could be the ideal ones to be controlled by the interacting molecules. Pure autocatalytic positive feedbacks could enforce collection of most of these autocatalytic molecules at one SPB, but that would not ensure that the other molecule type ends up at the other SPB (not shown). Thus the simplest way of implementing a positive feedback loop that can bring the two molecule types to the opposite SPBs should be based on a double-negative type positive feedback loop [25]. In such a minimal model molecule *X* removes molecule *Y* from the SPBs, while molecule *Y* induces the unbinding of molecule *X* (Fig. 1A). In this way both components remove their own inhibitor and with this they positively influence their own binding to the SPB. If *X* has a little bias at one of the SPBs it will remove all of *Y* from this place and help its own recruitment to this SPB. At the same time *Y* can pile up at the other SPB, since its inhibitor *X* was moved to the other SPB. Indeed *Y* speeds up the removal of *X* from this place and by this, speeds up the establishment of asymmetry. Computational simulation of such a minimal model shows that with a little noise in the initial amounts of *X* and *Y* at SPBs or a minimal (0.1%) bias in the binding rate to the old SPB is enough to induce asymmetry from a symmetric initial condition (Fig. 1B). The molecular interactions of Fig. 1A were translated into the computational model with a non-linear enzymatic reaction step for the action of *X* on *Y* unbinding (see Materials and Methods for details). Thus a model with antagonistic interactions of two molecule types, with (in biology often observed) non-linear kinetics can serve as a minimal model of asymmetry establishment between two SPBs.

### Minimal molecular network to drive asymmetry establishment

Next we investigated if we have any evidence for the existence of such an antagonistic, double-negative feedback loop among regulators of cytokinesis timing in fission yeast cells. The SIN can be considered as a linear pathway from Spg1 through Cdc7 and Sid1 activation, leading eventually to the recruitment and activation of Sid2 [6], [7]. The Cdc16-Byr4 complex inhibits Spg1 and as a result Cdc7 binding to the SPB, thus it is a negative regulator of SIN. It was also shown that Byr4 can bind to an SPB only if Cdc11 is fully dephosphorylated [26] and Sid2 is responsible for part of the phosphorylation on Cdc11 [20]. Cdc11 is known to be (at least partially) dephosphorylated by the SIN Inhibitory Phosphatase Complex SIP [21], which we also consider as a regulator of the proposed minimal system. In summary Cdc16-Byr4 inhibits SIN and SIN inhibits Cdc16-Byr4 localization to SPB, giving an antagonistic double-negative feedback loop (Fig. 1C). We can update the wiring diagram of Fig. 1A with the basics of the molecular details of this antagonistic interaction by joining the SIN members in a single variable and representing the Cdc16-Byr4 complex by its limiting component Byr4. The wiring has to be further extended as SIN is not directly inhibiting Byr4, but through phosphorylating Cdc11, which form cannot support Byr4 recruitment to SPB. Thus, instead of direct activation of Byr4 removal (as it is on Fig. 1A), SIN inhibits the facilitator of Byr4 binding (Fig. 1D). This adds an extra step in the system, but does not change the signs of the interactions proposed above.

**Figure 2:**
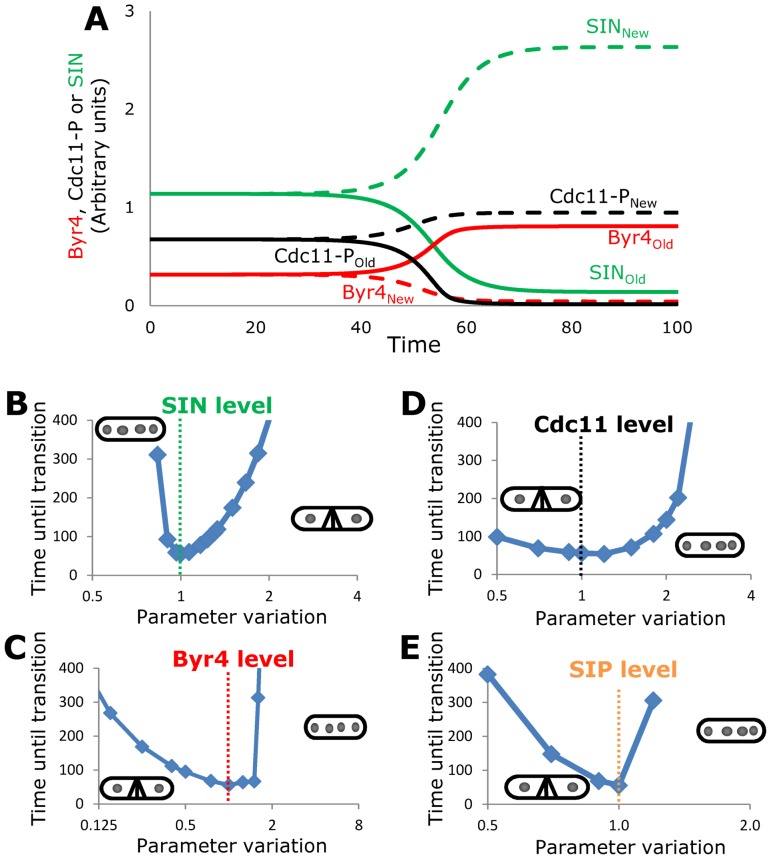
Behavior of the minimal molecular model of SIN asymmetry establishment. (**A**) A small bias in Byr4 binding to SPB is enough to establish asymmetry from an initial condition corresponding to metaphase-anaphase transition. Solid lines for molecules at old SPB, dashed lines for molecules at new SPB, time in arbitrary units. (**B–E**) Timing of transition (reaching the inflection point in the SIN_New_ curve) greatly depends on total level of each of the investigated proteins (plotted on a log_2_ scale). In each plot the basal (wild type) parameter is normalized to 1 (dashed lines) and the final phenotype of the effect of increase and decrease are noted with the multinucleate and multiseptate *S. pombe* cartoons. SIN level cannot be varied in either direction (A), Byr4 cannot be increased, while major reduction has also a deleterious effect. (B) Cdc11 and SIP can be changed also in small regimes (C,D). The observed multiseptate phenotype at reduced Cdc11 levels might come from the fact that we start simulations with an initial mitotic high SIN state, which might not be even reached in this mutant, while the multinucleate phenotype of Cdc11 overexpression contradicts literature data [10], [34].

This system can be also turned into a computational model and in this case we can move the non-linearity to the Cdc11 multistep phosphorylation-dephosphorylation reactions (captured by an appropriate non-linear function [24], [27], [28]). Simulation of this model shows that asymmetry of SIN can be established from an initial metaphase state (high SIN, low Byr4 at both SPBs). After the transition, the active SIN is localized together with phosphorylated Cdc11 to the new SPB, while Byr4 is at the old SPB with dephosphorylated Cdc11 (Fig. 2A). Cdc11 is not moving between the two SPBs, it just changes its phosphorylation state depending on the presence of regulators at a given SPB. To reach this asymmetry all we had to assume is that Byr4 has a 0.1% higher affinity to bind to the old SPB than to the new SPB. This (or a much higher) initial bias could come from inherited phosphorylated proteins that are specifically present at the old SPB [15].

It is known that proper cytokinesis greatly depends on the total amount of SIN components and its regulators [29], [30]. Overexpression of Spg1, the uppermost member of SIN leads to hyperactivation of SIN and to a multiseptated phenotype when cells periodically lay down septa without cleaving them [12]. A similar phenotype is observed when Cdc16, Byr4 or to some extent SIP function is lost [21], [31], [32]. On the other hand mutations in SIN components and Byr4 overexpression lead to SIN inactivation and to a multinucleate phenotype when septum formation and cell division is totally abolished [12], [14], [32]. We observe similar behavior in the simulations of the model if the total cellular levels of SIN and Byr4 are perturbed (Fig. 2B–E). SIN level can be changed only in a very narrow window, even very small changes lead to delays in asymmetry establishment and doubling or halving of the original amount already shows the experimentally observed terminal phenotypes (Fig. 2B). Byr4 cannot be increased either, small reductions do not lead to major delays in asymmetry but below a certain threshold the observed phenotype reveals (Fig. 2C). The simulated high sensitivity to Cdc11 levels (Fig. 2D) is contradicting the literature data as overexpression should not lead to a phenotype [10], while mutations in Cdc11 function should lead to multinucleate phenotype [33]. This latter problem comes from the fact that we initiate the model in late mitosis with high SIN levels, which cannot be reached in Cdc11 mutants as SIN binding to SPB requires Cdc11 function. Furthermore Cdc11 is also needed for the activity of downstream SIN components (Sid1, Sid2) [10]. A major extension of the model with the whole mitotic regulation of SIN could resolve this issue, here we keep our focus on asymmetry establishment after anaphase onset.

Overexpression of Csc1, a member of the SIP complex leads to multinucleate cells and some SIP mutant cells (*csc1Δ*) show multiple septa [21]. Although it is not clear if overexpression of one of the components of the SIP complex is enough to induce higher SIP phosphatase activity or if it has a dominant negative effect, the simulated high sensitivity to SIP levels (Fig. 2E) resembles experimental observations [21]. In summary the minimal molecular model of SIN asymmetry regulation properly simulates most experimental observations. The major failure of the model is on the high sensitivity to Cdc11 levels. The experimentally observed low sensitivity to Cdc11 overexpression [34] might be explained by a limiting effect of Sid4, which helps Cdc11 to recruit SIN members to SPB [35], but we can also investigate Cdc11 in more detail if we consider its different phosphorylation sites.

### Revealing the importance of the phosphorylation states of Cdc11

**Figure 3:**
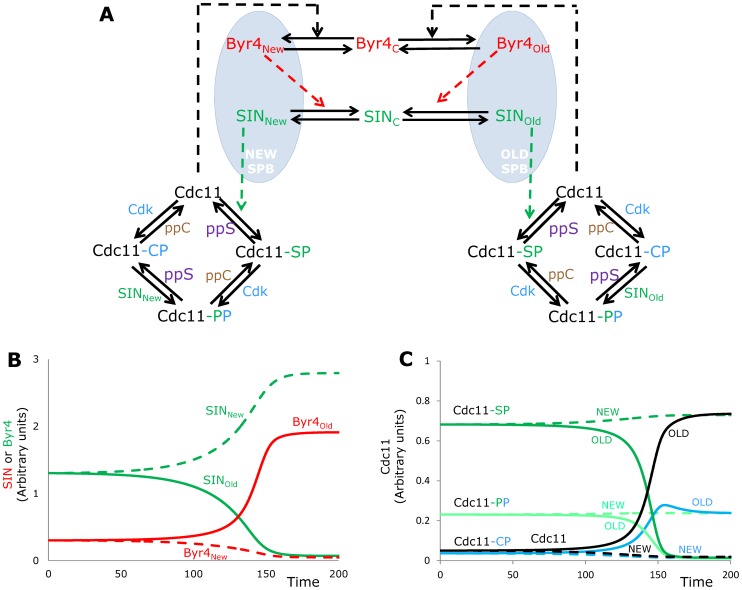
Model expansion on Cdc11 regulation. The minimal model was extended by multiple phosphorylation forms of Cdc11 (**A**). It can be phosphorylated by SIN (green), Cdk (light blue) and both. The Cdk sites are assumed to be dephosphorylated by the unknown phosphatase “ppC”, while the SIN sites are dephosphorylated by an unknown phosphatase, “ppS”. (**B**) Simulation time course of SIN and Byr4 activities at the two SPBs (solid for Old, dashed for New). (**C**) Changes in the various phosphorylated forms of Cdc11. Notations and color code on forms on panel A.

Cdc11 is known to be phosphorylated on multiple sites by SIN (specifically shown for Sid2 in [20]) but Cdc11 also contains Cdk phosphorylation sites [20], [35]. SIP was discovered as a SIN Inhibitory PP2A Phosphatase Complex as it can remove phosphate groups from Cdc11 [21]. PP2A complexes often counteract Cdk phosphorylations [36], so it could be that SIP is working on the Cdk phosphorylation sites of Cdc11 and either SIP or another phosphatase removes the phosphates from SIN sites. Furthermore, it was observed that removal of SIN phosphorylation sites from Cdc11 (mutating five serine to alanine) leads to advanced asymmetry establishment [20], which could not be captured by the minimal model. To overcome these issues we extended the model with Cdk phosphorylation of Cdc11 (Fig. 3A). Cdc11 can exist in at least four different forms: Cdk phosphorylated (Cdc11-CP), SIN phosphorylated (Cdc11-SP), phosphorylated by both (Cdc11-PP) and non-phosphorylated (Cdc11) and only this latest form can support Byr4 binding to SPBs. As we have no information on the target sites of SIP or other phosphatases acting on Cdc11 we investigate the effects of both dephosphorylation steps separately. We assume a hypothetical phosphatase *ppC* to remove phosphates from Cdk site, while another phosphatase *ppS* works on SIN sites (Fig. 3A). Similarly to the simple model above, SIN and Byr4 dynamics at the two SPBs follows the experimentally observed trend (Fig. 3B). The various forms of Cdc11 are converted into each other as cytokinesis proceeds, with ∼75% Cdc11 becoming dephosphorylated and 25% remaining Cdk phosphorylated at the old SPB (solid black line of Fig. 3C) and most of Cdc11 at the new SPB is phosphorylated mostly by SIN (dashed green on Fig. 3C).

**Figure 4:**
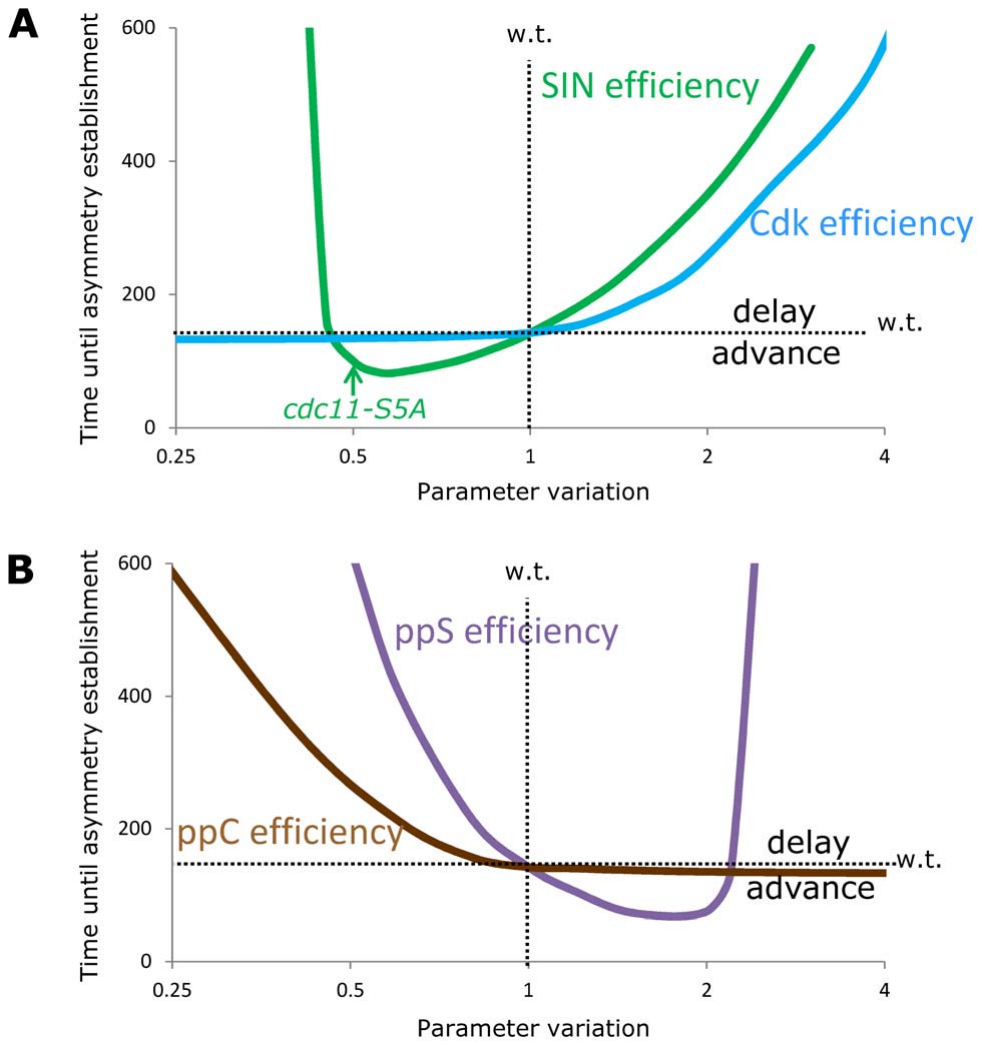
Sensitivity of asymmetry establishment timing on Cdc11 modification efficiencies. Efficiencies of SIN and Cdk phosphorylation (**A**) and ppC and ppS dephosphorylation (**B**) on the time it takes to reach asymmetry in SIN activity (inflection point in Byr4_Old_ curve). Small decrease in SIN efficiency on Cdc11 phosphorylation advances asymmetry (this is what was observed for the *cdc11-S5A* mutant, noted with a green arrow), while major decrease in this efficiency delays the transitions and eventually leads to high Byr4 (∼multinucleate) phenotype. Increase in this efficiency leads to SIN hyperactivation (∼multisetpate) phenotype. Decrease in Cdk efficiency has no major effect on asymmetry, but increase in this delays the transition and can lead to SIN hyperactivation. Increase in ppC seems to have no effect on asymmetry timing, while increase in ppS can lead to Byr4 hyperactivation. All wild type parameter values are normalized to 1, thus horizontal dotted lines show the wild type timing of asymmetry establishment.

This model is sensitive to changes in SIN and Byr4 levels (Fig. S1A,B) as the minimal model was (Fig. 2B,C), but now the sensitivity of Cdc11 overexpression and the simulated multinucleate phenotype of the minimal model (Fig. 2D) is lost, since Cdk can phosphorylate even high levels of Cdc11 and by this inhibit Byr4 binding to the Cdc11, which is present in excess (Fig. S1C). With these we fixed the simulations of the major phenotypes. Literature data suggest that the timing of asymmetry establishment is highly sensitive to the Cdc11 phosphorylation state [20]. Fig. 4 shows how perturbations in the SIN and Cdk phosphorylation efficiencies and in the phosphatase efficiencies of ppC and ppS affect the timing of asymmetry establishment in the detailed model. Small decreases in SIN efficiency advance asymmetry, while severely reduced SIN phosphorylation on Cdc11 leads to a multinucleate phenotype. Advances were observed for the Sid2 phosphorylation site removed *cdc11-S5A* mutant [20], which is matched with an approximate halving of SIN efficiency on Cdc11 (arrow on Fig. 4A). Since the phosphorylation of SIN on Cdc11 in the model captures all negative effects of SIN on Byr4 activation and the experimentally observed effect of SIN sites removal from Cdc11 can be captured by a partial reduction of this effect, suggesting that SIN has to phosphorylate other targets which are regulating Byr4 activity/localization (see details on this in the discussion). On the other hand, total reduction in Cdk phosphorylation efficiency has no effect on asymmetry timing, while an increase in the Cdk site phosphorylation, similar to high SIN efficiency led to serious delays and eventually to a multinucleate phenotype (Fig. 4A). Thus, Cdk mostly serves as an initiator of the Cdc11 phosphorylation state and it is not directly involved in asymmetry timing, but if Cdk (or SIN) phosphorylation on Cdc11 is constantly high then Byr4 cannot bind to SPBs and this leads to multinucleate phenotype.

Serious reduction in either hypothetic phosphatase activity leads to multinucleate phenotype, while milder reduction causes a delay. Interestingly increase in ppC efficiency (overexpression of the hypothetical phosphatase) does not cause any phenotype in the model, while ppS overexpression leads to multinucleate phenotype (Fig. 4B). If we assume that the overexpression of the SIP component, Csc1, induces higher SIP activity (if this is the only limiting factor in the complex) leading to the observed multinucleate phenotype [21], then the model predicts that SIP should have roles in removing phosphates catalyzed by Sid2 to Cdc11 (at least when it is overexpressed). Since other mitotic phosphatases, like the Cdc14 phosphatase, Clp1/Flp1 [37], [38] or the PP2A phosphatases Par1 and Pab1 [39], [40] have been associated with SIN function and recent results suggests a role for Clp1 in Cdc11 dephosphorylation [41], we cannot conclude on the exact role of SIP only by simulating single perturbations on Cdc11 phosphorylation.

### Predictions and experimental tests on double and triple mutants

**Figure 5:**
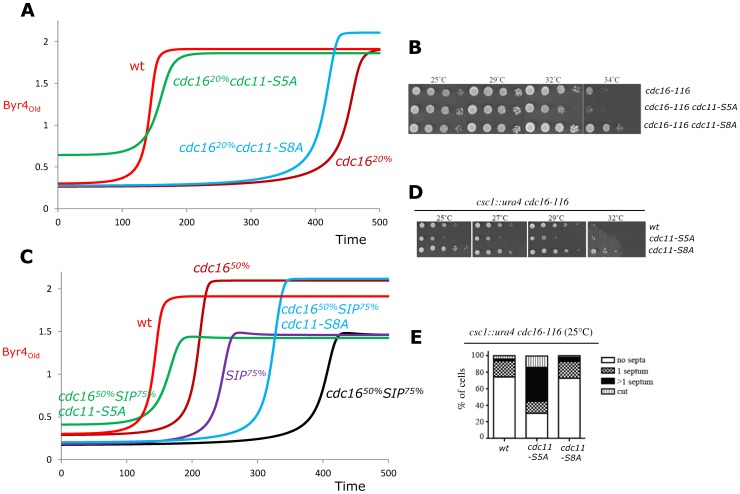
Predictions and experimental tests on collective effects of multiple mutations on SIN asymmetry establishment timing. (**A–C**) Simulations of interactions of *cdc16^ts^* (**A**) and *cdc16^ts^ sip^−^* (**C**) mutations with mutations in *cdc11* phosphorylation sites. Reduced level of Cdc16 activity was simulated by the indicated reduction in Byr4 efficiency on SIN inactivation. Mutations in SIP was captured by 25% reduction in both *ppC* and *ppS* efficiency. As shown on Fig. 4 we assume that 50% SIN efficiency corresponds to the *cdc11-S5A* mutation. Time courses of Byr4 level changes at the old SPB are plotted as a representative proxy of SIN asymmetry establishment (other variables follow this as on Fig. 3). (**B–D**) Spot assays: The indicated cultures were serially diluted and spotted on YES agar medium, and grown at the specified temperatures. (**B**) At 32°C *cdc11-S8A* can partially compensate effects of the temperature sensitive *cdc16-116* mutation, while *cdc11-S5A* makes it even more severe. (**D**) *cdc11-S5A* decreases while *cdc11-S8A* minimally increases viability of *cdc16^ts^ sip^−^* mutants. (**E**) Phenotypes observed in the colonies of panel D at 25°C. n>300 cells for each strain.

In our first double perturbation test we investigated the interactions between perturbations in SIN and Cdk efficiency on Cdc11 phosphorylation versus mutations in the Byr4 effector Cdc16 efficiency on SIN inactivation (Fig. 5A). Cdc16 mediates the GAP-activity that induces Spg1 inactivation and it is localized by Byr4 [13], thus mutations in Cdc16 can be simulated in our model by changing the efficiency of Byr4 on SIN inactivation (*k_Soff_* in Supplementary Text S1). The temperature sensitive *cdc16-116* mutant can proliferate at 25°C while at higher temperatures the activity of this mutant protein is gradually reduced and eventually the cells are unable to inactivate SIN leading to a multiseptated phenotype at 36°C [31]. Simulation of this mutant by setting Byr4 efficiency on SIN to 20% of the wild type value shows a strong delay in asymmetry establishment (Fig. 5A). The model predicts that this delay can be compensated for mildly by removal of Cdk phosphorylation sites from Cdc11 but very efficiently by the *cdc11-S5A* mutants of SIN phosphorylation on Cdc11 (Fig. 5A). To test this prediction first we used a Cdk site mutant version of Cdc11 [35] that substitutes the eight Cdk phosphorylation sites from Cdc11 [20] and tested its effects on cell viability. As reported previously [35], removal of Cdk phosphorylation sites from Cdc11 has no major effect on cell viability, matching the simulation results (Fig. 4A). The *cdc11-S8A* mutant could indeed mildly compensate for the defects of *cdc16-116* (Fig. 5B), while the SIN (Sid2) sites removed *cdc11-S5A* mutation instead of rescuing the phenotype rather exacerbated it (Fig. 5B).

It was shown that SIP phosphatase complex removes phosphate groups from Cdc11 and that mutations in SIP components give an additive effect to *cdc16* mutations [21]. To investigate the discrepancy between model and experiment further, we tested if *cdc11-S5A* and *cdc11-S8A* mutants can compensate this additive effect of SIP and *cdc16* mutations. First we simulated the *cdc16* mutation by reducing the effect of Byr4 on SIN to the half of the original value and the *csc1Δ* SIP mutation by setting both ppC and ppS to 75% of the wild type values. The simulations indeed match the additive effects of these mutations (Fig. 5C). Greater decreases lead to even greater delays in asymmetry establishment and eventually to a multiseptate phenotype (not shown). The simulations of cdc11 phosphosite mutants predict that major SIN sites removal (*cdc11-S5A*) can compensate the additive effect of SIP and Cdc16 quite well, while Cdk site removal has only minor compensatory effects (Fig. 5C). Experimental tests show that the double mutants of *cdc16-116* and *csc1Δ* is mildly compensated by Cdk phosphorylation sites removal from Cdc11, matching the prediction (Fig. 5D). At the same time the double mutant phenotype becomes more severe after Sid2 phosphorylation site removal (Fig. 5D). Phenotypic analysis of these cells show that the number of multiseptated and cut cells increased in the *cdc16-116 csc1Δ cdc11-S5A* triple mutants (Fig. 5E), suggesting that SIN might come too early and stays active longer in some of these cells.

The discrepancies between simulations and experimental results show that blocking Sid2 phosphorylation of Cdc11 has consequences other than allowing enhanced Byr4 binding to SPBs [26], furthermore, perturbation in the SIP phosphatase complex (*csc1Δ*) does not change the severe phenotype of *cdc16-116 cdc11-S5A* mutants. These, and other earlier findings [20], [21], [41] suggest that Sid2 phosphorylation might prime Cdc11 for dephosphorylation at other sites and Byr4 binding, making SIN an indirect activator of Byr4. Recent results suggest that such dephosphorylation events might be catalyzed by the Cdc14-like Clp1/Flp1 phosphatase, even in the absence of SIP activity [41]. Removal of both SIN and Cdk phosphorylation sites from Cdc11 (*cdc11-S13A*) does not have a major effect on cell viability, furthermore SIP activity still has an effect on the phosphorylation state of Cdc11 in *cdc11-S13A* cells [41], indicating that SIP dephosphorylates Cdc11 at sites modified by other kinases. Thus our findings, together with recent literature data, indicate that our understanding of Cdc11 regulation by phosphorylation-dephosphorylation events is incomplete.

### Simulations of peculiar observations on SIN activation/inactivation dynamics

**Figure 6:**
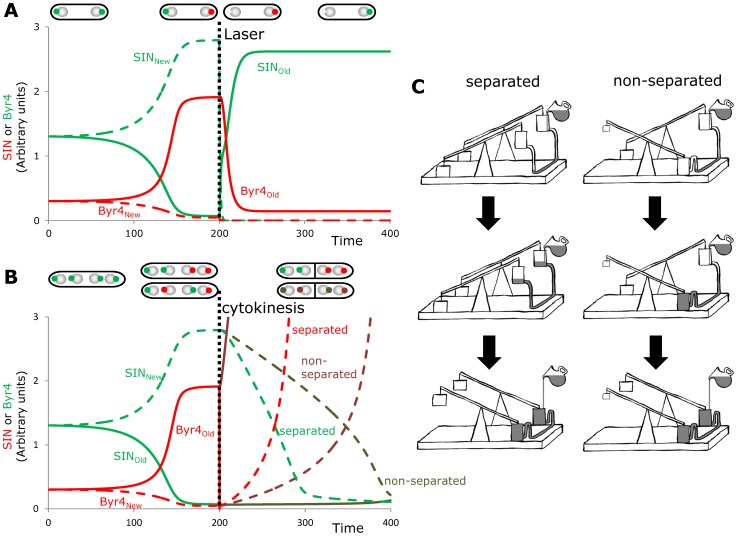
Simulations of the most peculiar observations in SIN asymmetry establishment. (**A**) We simulated the laser ablation of the new SPB after anaphase (top), what leads to SIN activation at the old SPB [42]. At 200 time steps (horizontal dotted line) we stopped transport towards the new SPB and let all its content diffuse into the cytoplasm. (**B**) Simulation of the termination of SIN activity. At 200 time steps we induced the production (or reduced degradation) of new Byr4 molecules (as a proxy for the unknown signal that turns off SIN). At the same time we cut the communication between the two SPBs as it happens at the end of cytokinesis (“separated”, lighter color curves) or let the two SPBs communicate through the cytoplasm as it happens in some dikarions [43] (Non-separated, darker color lines and dots on top panel). If the cells are separated the newly formed Byr4 goes to the only existing new SPB, while if the cells did not separate it will be constantly recruited to the old SPB, thus SIN at the new SPB will turn off much later. (**C**) Seesaw metaphors of the two cases of panel B (seesaws are common examples of antagonistic interactions with two opposing steady states). The right arm of the seesaws represent SIN activity at the two SPBs, and they are connected to each other (water can flow between them in the metaphor - molecules can diffuse between SPBs in cells). The situation where the active and inactive SPBs are separated is captured on the left, where both SPBs are active, water is poured in (signals induce SIN inactivation) they both can turn together. On the right (non-separated active and inactive SPB) one SPB has high SIN, the other has low SIN. When water is poured in, first it flows to the lower (already inactive SIN) bucket and the upper seesaw will turn only if the lower bucket and the pipe are full.

We have shown above that the model can capture the basic behavior of SIN mutants in asymmetry establishment and can accurately predict the behavior of some mutant combinations. There are a few, so far, unresolved experimental findings that ask for computational models to help understand them. Magidson et al. [42] found that if in anaphase, when SIN asymmetry is already established, the new SPB containing active SIN was ablated with a laser, then the SIN starts to get activated at the old SPB. To simulate this experiment we stopped the simulations when asymmetry was reached and uncoupled the new SPB from the rest of the cell. Fig. 6A shows that if some SIN from the ablated new SPB can fall back to the cytoplasm (or constantly produced there – not shown) then it can move to the old SPB and remove Byr4 activity there. This happens because the free cytoplasmic SIN now can start to bind to the only existing old SPB. Although this is slow at the beginning, as SIN starts to phosphorylate Cdc11, Byr4 cannot be as efficiently recruited anymore. As this positive feedback of SIN activation (through inhibiting the binding of its inhibitor) speeds up, more and more SIN gets to the only existing SPB and at the same time Byr4 is getting removed.

In another interesting experiment, by cleverly creating dikarions Garcia-Cortes and McCollum [43] investigated cells with four SPBs present at the time of mitosis. They found that when two SPBs with active SIN go to one daughter cell and two with inactive SPBs to the other, then cells separate properly and SIN gets inactivated right after division. In contrast, when both daughters inherit one active and one inactive SPB then the SIN could not turn off properly. We simulated these two scenarios by removing (separated) or maintaining (non-separated) the communication between the inactive, old SPB and the cytoplasm of the new SPB and followed the speed of SIN inactivation at the new SPB (Fig. 6B). To mimic the unknown factors that induce SIN inactivation after cell separation we started to increase the cytoplasmic Byr4 level in the cells. We followed this approach as in our small model Byr4 acts as the only inhibitor of SIN, but any other abrupt change in the SIN/Byr4 ratio as a result of cytokinesis would have a similar effect in the model. Although the exact mode of SIN inactivation after completion of cytokinesis is not clear, the simulation results show that the same inactivation strength lead to a much faster SIN inactivation when the two SPBs were separated (Fig. 6B). This happens, because in the separated case all inhibitors of SIN can start to work on the SPB with the active SIN, while in the non-separated case the newly produced inhibitors are still recruited to the already inactive SPB, thus they cannot reach the active SIN on the other SPB. A mechanical metaphor explains both situations on Fig. 6C. The antagonistic, double-negative feedback loop leads to situations when on one SPB SIN can always win against Byr4. If two or more SPBs are in the same cytoplasm then this antagonism leads to asymmetry establishment and strong maintenance of this state. These results suggest that cells are sensitive to SIN/Byr4 ratio before establishing the asymmetry, but once they established SIN asymmetry the strong antagonism can compensate small changes in the SIN/Byr4 balance. After communication between the daughter nuclei is halted by the septum, the balance is important again and the SIN-Byr4 antagonism can help the fast inactivation of SIN.

## Discussion

Asymmetric activation of the SIN on one of the two SPBs is a necessary feature of proper cell division timing in fission yeast cells [18], [19]. Similar asymmetry is established between the SPBs of the budding yeast *Saccharomyces cerevisiae* [44], [45]. In the case of such asymmetrically dividing organisms, the asymmetry establishment is better characterized [46] and mathematical modeling has already facilitated discoveries of the detailed mechanism [22]. Here we establish a minimal model to understand the major driving forces of symmetry breaking in SIN activity at the two SPBs in fission yeast. This minimal model is based on the antagonistic interaction of two molecules that are inhibiting each other's localization to the SPB (Fig. 1A). This system resembles the basic models of Notch-Delta antagonism that is used to model lateral inhibition [47]. Indeed the underlying dynamics in both cases leads to a pitchfork bifurcation ([23] and Fig. S2). The model behaves as an efficient switch [48], which brings one molecule type to one SPB and its antagonist to the other, with some remaining in the cytoplasm. In the case of SIN asymmetry establishment the clear candidates for such antagonistic interactions are the members of the SIN and its inhibitory complex Byr4-Cdc16. Byr4-Cdc16 inhibits SIN activity [13], while there is also some evidence that SIN indirectly inhibits Byr4 localization [20], [26]. Such antagonism is a special case of a positive feedback loop, where the two components cannot coexist, either one of them is winning and inhibiting the other [25]. In the case of SIN asymmetry establishment, the two antagonists are winning at different SPBs. Indeed when the new SPB is starting to get enriched in SIN, it means SIN has to drop a bit on the other SPB, which enables Byr4 to win on the old SPB. In this way SIN activation at one SPB helps Byr4 activation on the other SPB explaining some controversial observations which suggest that SIN components and mitotic phosphatases seem to activate both SIN and Byr4 [19]. Thus any signal that leads to the induction of asymmetry establishment basically activates SIN (at the new SPB) as well as Byr4 (at the old SPB). The major initiating step is the drop in Cdk activity in anaphase in parallel with spindle elongation that moves the SPBs far apart. Our simulations are initiated exactly at this step. Possible spatial extensions of the model might reveal some role for SPB positioning, although the quick turnover of active Sid2 [20] might rule out any major effect of space in SIN asymmetry establishment.

A crucial point here is that such a system with an antagonistic switch works properly only if the total amounts of the two antagonists are present in a given ratio (1 in our case, but this value is determined by the exact rate constants), any perturbation of this balance can lead to a situation where either SIN or Byr4 wins on both SPBs. Indeed fission yeast cells are very sensitive to the overexpression of either Byr4 or the SIN limiting factor Spg1, but the joint overexpression of these two can be greatly tolerated by the cells [30] suggesting that indeed their ratio is important for proper asymmetry establishment. The model suggests that once the asymmetry is established this balance is not that crucial anymore, but later the same antagonism can help the fast inactivation of SIN after septation. At this stage only the new SPB inheriting daughter has active SIN signaling, but this is turned off for an unknown signal that most probably flips the SIN/Byr4 balance.

The extended minimal model (Fig. 3A) is still a simplification of the whole system of SIN regulation as here we concentrated only on the interactions that are important for the asymmetry establishment in SIN activity (see [4] for a model on SIN activation timing). Still this simple model was able to capture qualitatively multiple experimental results on single molecule perturbations (Fig. 2B–E and Fig. S1), explain results of experiments when the number of SPBs were perturbed in the cells (Fig. 6) and predict the behavior of some double and triple mutants (Fig. 5). The prediction on the compensatory effects of Cdk sites removal from Cdc11 in a *cdc16* and *cdc16-116 csc1Δ* mutants were verified experimentally (Fig. 5A,B), the additive effects of SIP and Cdc16 mutants were also properly simulated, but the predictions on the double and triple mutants with *cdc11-S5A* failed (Fig. 5C–E). The *cdc11-S5A* mutation amplified the phenotype of *cdc16* and *cdc16-116 csc1Δ* mutants instead of compensating them. This does not mean that the model is totally wrong; it rather means that there is a hole in our knowledge about the backup mechanisms that regulate SIN activity when some of the major players are perturbed. Cdc11 is likely phosphorylated by other kinases (perhaps Cdc7 [26]) and proteomics screens found Clp1/Flp1 as a phosphatase acting on Cdk sites on Cdc11 [41], adding extra layers to the interaction system. Another possibility is that the Cdc11 phosphomutants may not recapitulate the result of asymmetric loss of phosphorylation in which only one SPB is affected and/or the investigated mutant combinations show a phenotype that is a result of other functions of Cdc16 [49]. Furthermore, it was earlier proposed that Clp1 might form another positive feedback loop with the SIN [19], [50], which could also play a role in the robustness of SIN asymmetry establishment. The proposed core mechanism of antagonistic interactions between activators and inhibitors of SIN should hold in all cases, just the main players might change as kinases and phosphatases as well as their target molecules might be perturbed in various mutants. There could be several other layers, where SIN and Byr4 antagonistically interact, as many other SIN regulators are targets of Cdk, SIN and Polo kinase dependent phosphorylation events [19]. A related prediction of the model is that SIN components have to act on other Byr4 regulator targets than Cdc11, as we could match the SIN phosphorylation sites removed *cdc11-S5A* phenotype only with a reduced efficiency of SIN, not with the total abolishment of this effect (Fig. 4A). The simplest possible solution would be if one of the SIN components could directly phosphorylate and by this mechanism inactivate Byr4. Since Byr4 has several candidate phosphorylation sites [29], [51] we cannot rule out this possibility.

The modeling results also predicted and the experiments verified that Cdk phosphorylation on Cdc11 is not a major factor in asymmetry establishment (Fig. 5A), it might rather play a role in setting up the initial state in early mitosis, when the top components of the SIN pathway are bound to both SPBs and Byr4 is removed from there. Interestingly, all of our simulation results show that in the initial mitotic state Byr4 is not totally absent from SPBs. This assumption on the initial conditions we needed to take to be able to achieve a fast asymmetry establishment. If Byr4 is completely absent from both SPBs in mitosis then it would be difficult for Byr4 to appear at one SPB in sufficient amounts (as it is sent away by active SIN) to turn on the positive feedback loop and establish asymmetry. Since Byr4 is a low abundance protein, it is hard to visualize [29], but the model suggests that even in mitosis some Byr4 might be localized at both SPBs.

It is still unknown what signal(s) turns off SIN activity in the daughter inheriting the new SPB after the completion of cytokinesis. The model of SIN and Byr4 antagonistic interactions successfully simulated the experimental results, which have shown that SIN activity can take over Byr4 at the old SPB if the new SPB was laser ablated before cell division ([42] and Fig. 6A) and it could also explain why SIN has a harder time to turn off when the two spindle pole bodies remain in the same cell after cell division ([43] and Fig. 6B). As we do not have information on the molecular details of the trigger that induces SIN inactivation in the daughter cell that inherited the SPB with active SIN, we needed to make a simple assumption that Byr4 production speeds up at this point, alternatively Byr4 degradation slows down when the daughters get separated [29]. Inactivation of SIN might happen even with a minor increase in Byr4 level, since once the old SPB is not in the same cytoplasm anymore it cannot serve as a sink for Byr4, thus Byr4 can pile up at the daughter with the active SIN and eventually turn SIN off. The prerequisite for this mechanism to work is a very fast turnover of Byr4, which has been suggested [29]. This and many other questions on the detailed regulation of SIN signaling still need to be addressed and as we have shown here, the system level view and computational modeling of the network can help our understanding and guide experimental discoveries. Here we could reach predictions on a semi-quantitative fashion (e.g.: what happens earlier/later in various mutants), measurements on molecular levels of the regulators and kinetic contacts of the reactions will enable the development of quantitative models that contain all molecular details of SIN activity regulation.

## Materials and Methods

### Model development

The wiring diagrams of Fig. 1A, 1D, 3A were converted into systems of ordinary differential equations (ODEs). Parameters of the models were identified by fitting their qualitative behavior to experimental observations. Molecular concentrations defined in arbitrary units. Future measurements of molecular levels could be used to convert the inferred parameter values to real biologically meaningful reaction rates. We assume fast diffusion between SPBs until cell separation cuts communication between SPBs. Parameter values, initial conditions and equations can be found in the Supplementary Text S1. Equations were numerically solved and simulated by the freely available software WINPP (http://www.math.pitt.edu/~bard/xpp/xpponw95.html).

### Experimental procedures

*S. pombe* strains were grown in yeast extract (YE) medium. Strain construction was accomplished through standard methods. The relevant genotypes and strain numbers used in this study were *cdc16-116 cdc11-S5A-GFP::kanR* (KGY1411), *cdc16-116 cdc11-GFP::kanR* (KGY3342), *cdc16-116 cdc11-S8A-GFP::kanR* (KGY8684), *cdc16-116 cdc11-GFP::kanR csc1::ura4^+^* (KGY12982), *cdc16-116 cdc11-S5A-GFP::kanR csc1::ura4^+^* (KGY12982), and *cdc16-116 cdc11-S8A-GFP::kanR csc1::ura4^+^* (KGY12984).

## Supporting Information

Figure S1 **Dependence of timing of asymmetry establishment on total protein levels in the extended minimal model of** **Figure 3A** **.** Similar figures as figure 2B–D for the more complex model. SIN dependence looks the same as in the minimal model just here the wild type behavior is not at the minimal time to reach asymmetry (**A**). Byr4 is similarly sensitive for reduction and for small increases as before (Fig. 2C), just here at higher values the time to asymmetry is advanced and eventually at a rate ∼2.5 times wild type the initial early mitotic state contains higher amount of Byr4 than SIN, thus these cells might not be able to perform the earliest steps of SIN activation (**B**). Cdc11 is now insensitive for overexpression, while its removal causes again a perturbed initial mitotic state, which cannot support high SIN activity in early mitosis (**C**).
(PDF)

Figure S2 **Symmetric steady state solutions for SIN levels at the two SPBs in the minimal model of SIN asymmetry establishment show that asymmetry emerges through a pitchfork bifurcation.** Stable (solid lines) and unstable (dashed) steady states of SIN activity at the old or new SPB. The two solutions totally overlap as the system is fully symmetrical. The calculations were performed with *kbias* = 0 to keep the system symmetric. Steady state solutions were calculated by Oscill8 (http://sourceforge.net/projects/oscill8/).
(PDF)

Text S1 **Description of parameters and variables of each model, together with equations, initial conditions and parameter values.**
(PDF)
