# Supplementary material for: Dynamics of SIN Asymmetry Establishment
Source: PLoS Comput Biol. 2013 Jul 11;9(7):e1003147. doi: 10.1371/journal.pcbi.1003147 (PMC3708865; doi:10.1371/journal.pcbi.1003147)
Supplement: Figure S1 — Dependence of timing of asymmetry establishment on total protein levels in the extended minimal model of Figure 3A . Similar figures as figure 2B–D for the more complex model. SIN dependence looks the same as in the minimal model just here the wild type behavior is not at the minimal time to reach asymmetry (A). Byr4 is similarly sensitive for reduction and for small increases as before (Fig. 2C), just here at higher values the time to asymmetry is advanced and eventually at a rate ∼2.5 times wild type the initial early mitotic state contains higher amount of Byr4 than SIN, thus these cells might not be able to perform the earliest steps of SIN activation (B). Cdc11 is now insensitive for overexpression, while its removal causes again a perturbed initial mitotic state, which cannot support high SIN activity in early mitosis (C). (PDF) [file pcbi.1003147.s001.pdf]

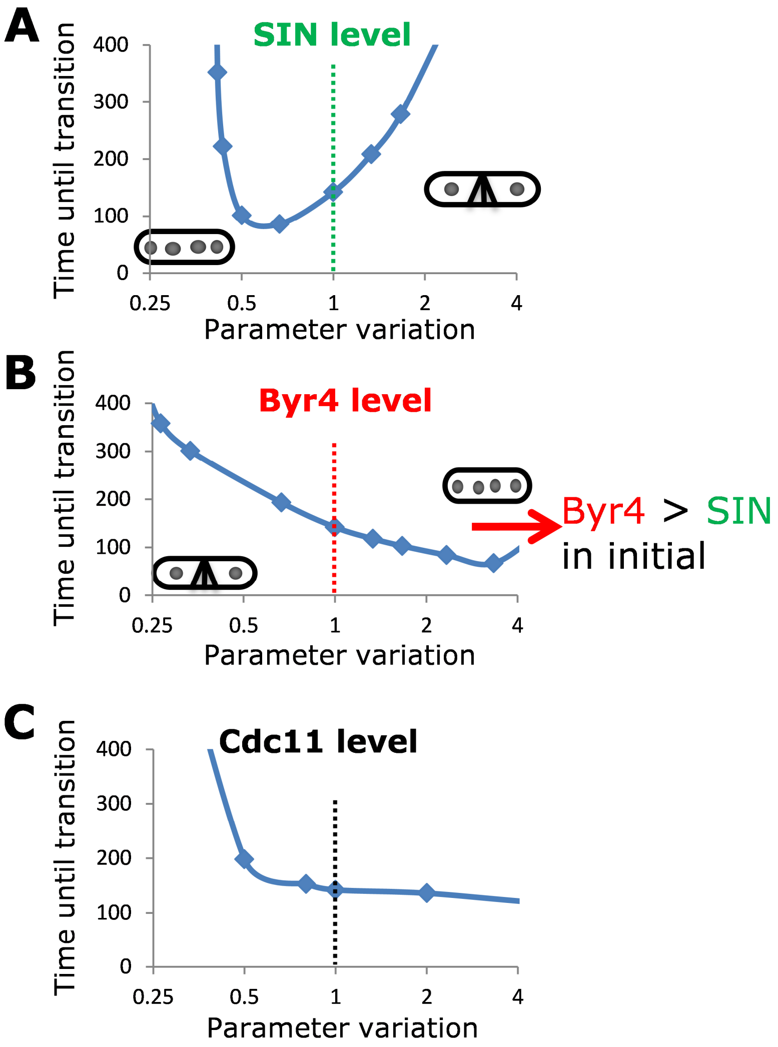

Figure S1. Dependence of timing of asymmetry establishment on total protein levels in the detailed model of Figure 3A. Similar figures as figure 2B-D for the more complex model. SIN dependence looks the same as in the minimal model just here the wild type behavior is not at the minimal time to reach asymmetry (**A**). Byr4 is similarly sensitive for reduction and for small increases as before (Fig. 2C), just here at higher values the time to asymmetry is advanced and eventually at a rate ~2.5 times wild type the initial early mitotic state contains higher amount of Byr4 than SIN, thus these cells might not be able to perform the earliest steps of SIN activation (**B**). Cdc11 is now insensitive for overexpression, while its removal causes again a perturbed initial mitotic state, which cannot support high SIN activity in early mitosis (**C**).
