# Supplementary material for: Dynamics of SIN Asymmetry Establishment
Source: PLoS Comput Biol. 2013 Jul 11;9(7):e1003147. doi: 10.1371/journal.pcbi.1003147 (PMC3708865; doi:10.1371/journal.pcbi.1003147)
Supplement: Figure S2 — Symmetric steady state solutions for SIN levels at the two SPBs in the minimal model of SIN asymmetry establishment show that asymmetry emerges through a pitchfork bifurcation. Stable (solid lines) and unstable (dashed) steady states of SIN activity at the old or new SPB. The two solutions totally overlap as the system is fully symmetrical. The calculations were performed with kbias = 0 to keep the system symmetric. Steady state solutions were calculated by Oscill8 (http://sourceforge.net/projects/oscill8/). (PDF) [file pcbi.1003147.s002.pdf]

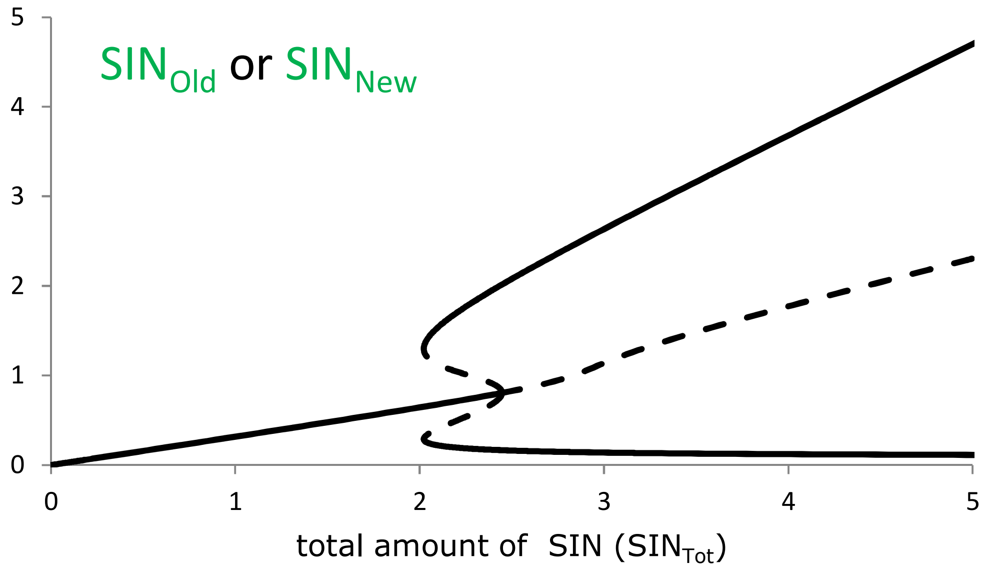

Figure S2. Symmetric steady state solutions for SIN levels at the two SPBs in the minimal model of SIN asymmetry establishment show that asymmetry emerges through a pitchfork bifurcation. Stable (solid lines) and unstable (dashed) steady states of SIN activity at the old or new SPB. The two solutions totally overlap as the system is fully symmetrical. The calculations were performed with *kbias* = 0 to keep the system symmetric. Steady state solutions were calculated by Oscill8 (http://sourceforge.net/projects/oscill8/).
