# Supplementary material for: Dynamics of SIN Asymmetry Establishment
Source: PLoS Comput Biol. 2013 Jul 11;9(7):e1003147. doi: 10.1371/journal.pcbi.1003147 (PMC3708865; doi:10.1371/journal.pcbi.1003147)
Supplement: Text S1 — Description of parameters and variables of each model, together with equations, initial conditions and parameter values. (PDF) [file pcbi.1003147.s003.pdf]

Supplementary Text S1

Dynamics of SIN asymmetry establishment

Archana Bajpai[1], Anna Feoktistova[2], Jun-Song Chen[2], Dannel McCollum[3], Masamitsu Sato[4], Rafael E. Carazo-Salas[5], Kathleen L. Gould[2], Attila Csikász-Nagy[1,6,7]

[1]The Microsoft Research-University of Trento Centre for Computational Systems Biology, Piazza Manifattura 1, Rovereto, 38068, Italy
[2]Howard Hughes Medical Institute and Department of Cell and Developmental Biology, Vanderbilt University, Nashville, TN 37212, USA
[3]Department of Microbiology and Physiological Systems and Program in Cell Dynamics, University of Massachusetts Medical School, Worcester, Massachusetts 01605, USA
[4]Department of Biophysics and Biochemistry, University of Tokyo, Tokyo, 113-0032, Japan and Department of Life Science and Medical Bioscience, Waseda University, Tokyo, 162-8480, Japan
[5]The Gurdon Institute, University of Cambridge, Cambridge, CB2 1QN, United Kingdom
[6]Department of Computational Biology, Research and Innovation Center, Fondazione Edmund Mach, San Michele all'Adige-38010, Italy
[7]Randall Division of Cell and Molecular Biophysics and Institute for Mathematical and Molecular Biomedicine, King's College London, London, SE1 1UL, United Kingdom

Table of Contents:

A TOY MODEL OF ASYMMETRY ESTABLISHMENT.. 2

A MINIMAL MODEL FOR SIN ASYMMETRY ESTABLISHMENT 4

EXTENDED MINIMAL MODEL OF SIN ASYMMETRY ESTABLISHMENT 6

A TOY MODEL OF ASYMMETRY ESTABLISHMENT

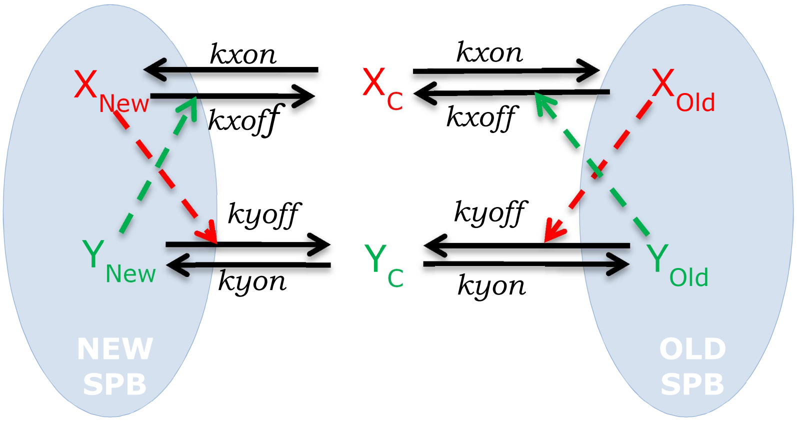

Wiring diagram of the toy model of asymmetry establishment. Same as Fig.1A, but with parameter names included.

Parameters of the toy model

Parameter Name	Description	values
kxon	Binding rate of X to SPBs	0.8
kxoff	Unbinding rate of X from SPBs	0.1
kyon	Binding rate at of Y to SPBs	0.5
kyoff	Unbinding rate of Y from SPBs	0.7
kbias	Initial bias to break symmtery	0.00001
J11	Michaelis constant of Y regulation	0.02
Xtot	Total level of X	2
Ytot	Total level of Y	3

Variables and their initial values

Variable Name	Description	Initial Value
$X_{Old/New}$	X bound to SPBs	0.94
$Y_{Old/New}$	Y bound to SPBs	0.85

MATHEMATICAL FORMULATION OF THE TOY MODEL OF ASYMMETRY ESTABLISHMENT

$dX_{Old}/dt = (kbias + kxon) \times X_c - kxoff \times Y_{Old} \times X_{Old}$

$dX_{New}/dt = kxon \times X_c - kxoff \times Y_{New} \times X_{New}$

$dy_{Old} / dt = kyon \times Y_c - ((kyoff \times X_{Old} \times Y_{Old}))/(J11 + Y_{Old})$

$dy_{New} / dt = kyon \times Y_c - ((kyoff \times X_{New} \times Y_{New}))/(J11 + Y\_New)$

Conservation Laws for the calculation of cytoplasmic forms Y_C and X_C.

$X_C = X_{tot} - X_{New} - X_{Old}$

$Y_C = Y_{tot} - Y_{New} - Y_{Old}$

A MINIMAL MODEL FOR SIN ASYMMETRY ESTABLISHMENT

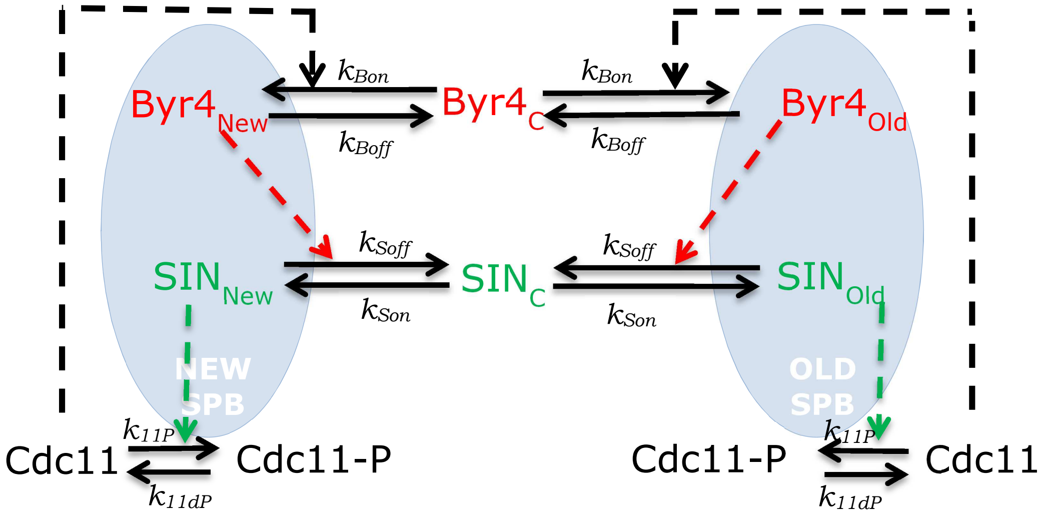

Wiring diagram of a minimal molecular network model that drive SIN asymmetry establishment. Same as Fig.1D, but with parameter names included.

Parameters of the minimal model

Paramters Name	Description	values
k_{Son}	Binding rate of SIN to SPBs	0.5
k_{Soff}	Unbinding rate of SIN from SPBs	1
k_{Bon}	Binding rate of Byr4 to SPBs	0.5
k_{Boff}	Unbinding rate of Byr4 from SPBs	0.7
kbias	Initial bias to break symmtery	0.00001
k_{11P}	Phosphorylation rate of Cdc11	0.4
k_{11dP}	Dephosphorylation rate of Cdc11	0.4
Js1	Michaelis constant of Cdc11 phsophorylation	0.1
$cdc11New_T = cdc11Old_T$	Total level of Cdc11 at both SPBs	1
$Byr4_{tot}$	Total level of Byr4	2
SIN_{tot}	Total level of SIN	3

Variables and their initial values

Variable Name	Description	Initial value

$SIN_{Old/New}$	SIN bound to SPBs	1.13
$Byr4_{Old/New}$	Byr4 bound to SPBs	0.31
$cdc11_{OldP/NewP}$	Phosphorylated forms of Cdc11	0.67

MATHEMATICAL FORMULATION OF THE MINIMAL MODEL OF ASYMMETRY ESTABLISHMENT

$$\frac{\mathrm{dSIN_{Old}}}{\mathrm{dt}} = k_{Son} \times \mathrm{SINc} - \mathrm{k}_{Soff} \times \mathrm{Byr4_{Old}} \times \mathrm{SIN_{Old}}$$

$$\frac{\mathrm{dSIN_{New}}}{\mathrm{dt}} = k_{Son} \times \mathrm{SINc} - \mathrm{k}_{Soff} \times \mathrm{Byr4_{New}} \times \mathrm{SIN_{New}}$$

$$\frac{\mathrm{dByr4_{Old}}}{\mathrm{dt}} = (\mathrm{kbias} + k_{Bon} \times \mathrm{cdc11_{Old}}) \times \mathrm{Byr4c} - k_{Boff} \times \mathrm{Byr4_{Old}}$$

$$\frac{\mathrm{dByr4_{New}}}{\mathrm{dt}} = (k_{Bon} \times \mathrm{cdc11_{New}}) \times \mathrm{Byr4c} - k_{Boff} \times \mathrm{Byr4_{New}}$$

$$\frac{\mathrm{dcdc11_{OldP}}}{\mathrm{dt}} = \frac{k_{11P} \times \mathrm{SIN_{Old}} \times \mathrm{cdc11_{Old}}}{\mathrm{Js1} + \mathrm{cdc11_{Old}}} - \frac{k_{11dP} \times \mathrm{cdc11_{OldP}}}{\mathrm{Js1} + \mathrm{cdc11_{OldP}}}$$

$$\frac{\mathrm{dcdc11_{NewP}}}{\mathrm{dt}} = \frac{k_{11P} \times \mathrm{SIN_{New}} \times \mathrm{cdc11_{New}}}{\mathrm{Js1} + \mathrm{cdc11_{New}}} - \frac{k_{11dP} \times \mathrm{cdc11_{NewP}}}{\mathrm{Js1} + \mathrm{cdc11_{NewP}}}$$

Conservation Laws for the calculation of cytoplasmic forms $Byr4_C$ and SIN_C and non-phosphorylated forms of Cdc11

$$\mathrm{Byr4c} = \mathrm{Byr4tot} - \mathrm{Byr4_{New}} - \mathrm{Byr4_{Old}}$$

$$\mathrm{SINc} = \mathrm{SINtot} - \mathrm{SIN_{New}} - \mathrm{SIN_{New}}$$

$$\mathrm{cdc11_{Old}} = \mathrm{cdc11oldt} - \mathrm{cdc11_{OldP}}$$

$$\mathrm{cdc11_{New}} = \mathrm{cdc11newt} - \mathrm{cdc11_{OldP}}$$

EXTENDED MINIMAL MODEL OF SIN ASYMMETRY ESTABLISHMENT

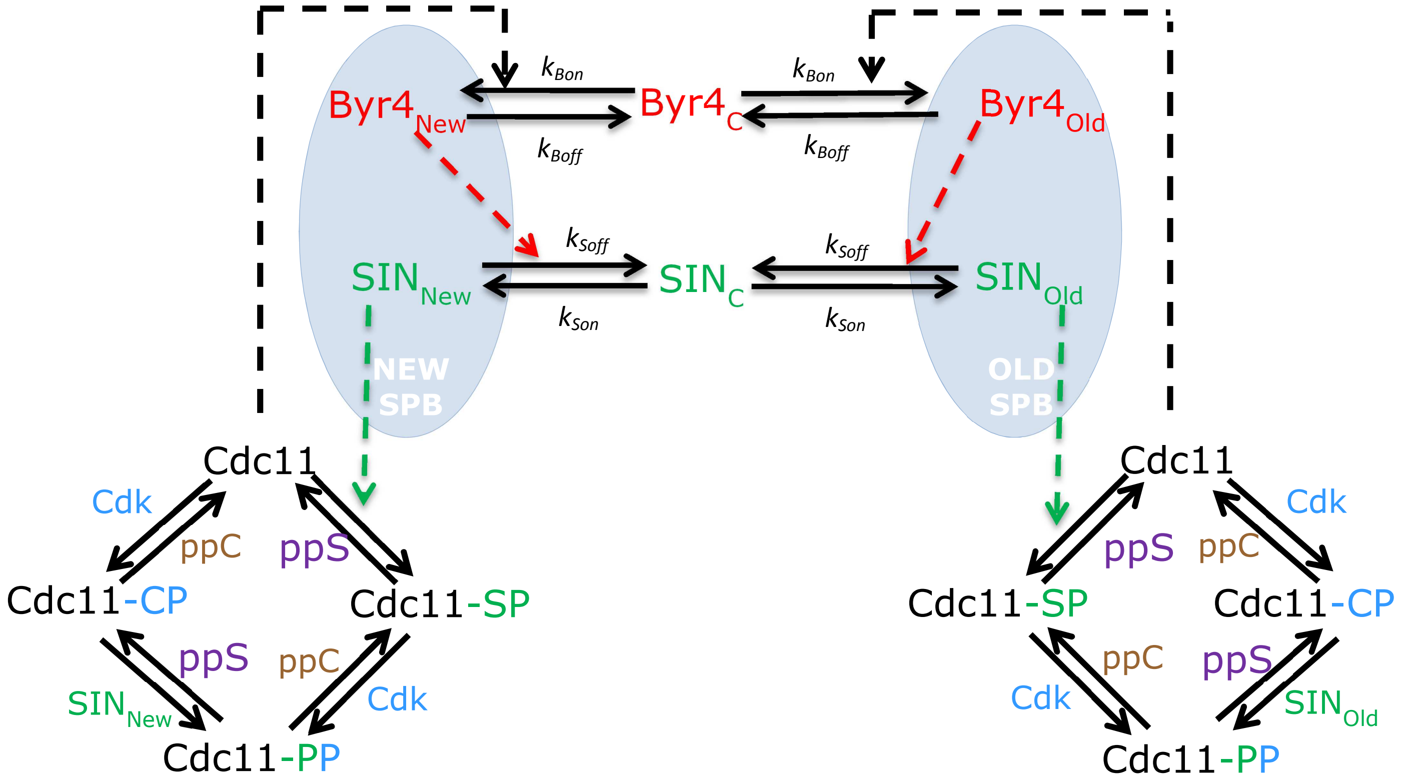

Wiring diagram of the extended minimal molecular network model that drive SIN asymmetry establishment. Same as Fig.3A, with parameter names included.

Parameters of the extended model

Paramters Name	Description	values
k_{Son}	Binding rate of SIN to SPBs	0.6
k_{Soff}	Unbinding rate of SIN from SPBs	0.6
k_{Bon}	Binding rate of Byr4 to SPBs	0.5
k_{Boff}	Unbinding rate of Byr4 from SPBs	0.2
kbias	Initial bias to break symmtery	0.00001
k11P	Phosphorylation rate of Cdc11 at SIN sites (SIN efficiency on Fig 4A)	0.2
kcdk	Phosphorylation rate of Cdc11 at CDK sites (Cdk efficiency on Fig. 4A)	0.08
ppC	Dephosphorylation rate of Cdc11 on Cdk sites (ppC efficiency on Fig. 4B)	0.1
ppS	Dephosphorylation rate of Cdc11 on SIN sites (ppS efficiency on Fig. 4B)	0.1
J11	Michaelis constant of Cdc11 phsophorylation	0.1
$Cdc11New/Old_{T}$	Total level of Cdc11	1
$Byr4_{tot}$	Total level of Byr4	3
SIN_{tot}	Total level of SIN	3

Variables and their initial values

Variable Name	Description	Initial value
$SIN_{Old/New}$	SIN bound to SPBs	1.30
$Byr4_{Old/New}$	Byr4 bound to SPBs	0.30
$Cdc11_{Old/New\ SP}$	Phosphorylated form of Cdc11 by SIN	0.68
$Cdc1_{Old/new\ PP}$	double phosphorylated form of Cdc11 by SIN and Cdk	0.23
$Cdc11_{Old/new\ CP}$	Phosphorylated form of Cdc11 by Cdk	0.03

MATHEMATICAL FORMULATION OF THE EXTENDED MODEL OF ASYMMETRY ESTABLISHMENT

$$\frac{\mathbf{dSIN_{Old}}}{\mathbf{dt}} = \mathrm{k}_{Son} \times \mathrm{SINc} - \mathrm{k}_{Soff} \times \mathrm{Byr4_{old}} \times \mathrm{SIN_{Old}}$$

$$\frac{\mathbf{dSIN_{New}}}{\mathbf{dt}} = \mathrm{k}_{Son} \times \mathrm{SINc} - \mathrm{k}_{Soff} \times \mathrm{Byr4_{New}} \times \mathrm{SIN_{New}}$$

$$\frac{\mathbf{dByr4_{Old}}}{\mathbf{dt}} = (\mathrm{kbias} + \mathrm{k}_{Bon} \times \mathrm{c11_{Old}}) \times \mathrm{Byr4c} - \mathrm{k}_{Boff} \times \mathrm{SIN_{Old}} \times \mathrm{Byr4_{old}}$$

$$\frac{\mathbf{dByr4_{New}}}{\mathbf{dt}} = (\mathrm{k}_{Bon} \times \mathrm{c11_{New}}) \times \mathrm{Byr4c} - \mathrm{k}_{Boff} \times \mathrm{SIN_{New}} \times \mathrm{Byr4_{New}}$$

$$\frac{\mathbf{dc11_{OldCP}}}{\mathbf{dt}} = (\mathrm{kcdk} \times \mathrm{c11_{Old}})/(\mathrm{J11} + \mathrm{c11_{Old}}) - (\mathrm{ppC} \times \mathrm{c11_{OldCP}})/(\mathrm{J11} + \mathrm{c11_{OldCP}})$$
$$-(\mathrm{k11P} \times \mathrm{SIN_{New}} \times \mathrm{c11_{OldCP}})/(\mathrm{J11} + \mathrm{c11_{OldCP}}) - (\mathrm{ppS} \times \mathrm{c11_{OldPP}})/(\mathrm{J11} + \mathrm{c11_{OldPP}})$$

$$\frac{\mathbf{dc11_{NewCP}}}{\mathbf{dt}} = (\mathrm{kcdk} \times \mathrm{c11_{New}})/(\mathrm{J11} + \mathrm{c11_{New}}) - (\mathrm{ppC} \times \mathrm{c11_{NewCP}})/(\mathrm{J11} + \mathrm{c11_{NewCP}})$$
$$-(\mathrm{k11P} \times \mathrm{SIN_{New}} \times \mathrm{c11_{NewCP}})/(\mathrm{J11} + \mathrm{c11_{NewCP}}) - (\mathrm{ppS} \times \mathrm{c11_{NewPP}})/(\mathrm{J11} + \mathrm{c11_{NewPP}})$$

$$\frac{\mathbf{dc11_{OldPP}}}{\mathbf{dt}} = (\mathrm{k11P} \times \mathrm{SIN_{Old}} \times \mathrm{c11_{OldCP}})/(\mathrm{J11} + \mathrm{c11_{OldCP}}) - (\mathrm{ppC} \times \mathrm{c11_{OldPP}})/(\mathrm{J11} + \mathrm{c11_{OldPP}})$$
$$+(\mathrm{kcdk} \times \mathrm{c11_{OldS}})/(\mathrm{J11} + \mathrm{c11_{OldS}}) - (\mathrm{ppS} \times \mathrm{c11_{OldPP}})/(\mathrm{J11} + \mathrm{c11_{OldPP}})$$

$$\frac{\mathbf{dc11_{NewPP}}}{\mathbf{dt}} = (\mathrm{k11P} \times \mathrm{SIN_{New}} \times \mathrm{c11_{NewCP}})/(\mathrm{J11} + \mathrm{c11_{NewCP}}) - (\mathrm{ppC} \times \mathrm{c11_{NewPP}})/(\mathrm{J11} + \mathrm{c11_{NewPP}})$$
$$+(\mathrm{kcdk} \times \mathrm{c11_{NewS}})/(\mathrm{J11} + \mathrm{c11_{NewS}}) - (\mathrm{ppS} \times \mathrm{c11_{NewPP}})/(\mathrm{J11} + \mathrm{c11_{NewPP}})$$

$$\frac{\mathbf{dc11_{NewS}}}{\mathbf{dt}} = (\mathrm{ppC} \times \mathrm{c11_{NewPP}})/(\mathrm{J11} + \mathrm{c11_{NewCP}}) - (\mathrm{ppS} \times \mathrm{c11_{NewS}})/(\mathrm{J11} + \mathrm{c11_{NewPP}})$$
$$-\mathrm{kcdk} \times \mathrm{c11_{NewS}})/(\mathrm{J11} + \mathrm{c11_{NewS}}) + (\mathrm{k11P} \times \mathrm{SIN_{New}} \times \mathrm{c11_{New}})/(\mathrm{J11} + \mathrm{c11_{New}})$$

$$\frac{\mathbf{dc11_{OldS}}}{\mathbf{dt}} = (\mathrm{ppC} \times \mathrm{c11_{OldPP}})/(\mathrm{J11} + \mathrm{c11_{OldCP}}) - (\mathrm{ppS} \times \mathrm{c11_{OldS}})/(\mathrm{J11} + \mathrm{c11_{OldPP}})$$
$$-(\mathrm{kcdk} \times \mathrm{c11oldS})/(\mathrm{J11} + \mathrm{c11_{OldS}}) + (\mathrm{k11P} \times \mathrm{SIN_{Old}} \times \mathrm{c11_{Old}})/(\mathrm{J11} + \mathrm{c11_{Old}})$$
